# Dynamic Neurocognitive Adaptation associated cognitive functions and cortical thickness in cognitively normal, mild cognitive impairment, and Alzheimer's Disease patients

**DOI:** 10.1002/alz70858_107103

**Published:** 2025-12-25

**Authors:** Filippo Cieri, Chad L. Cross, Pavithran Pattiam Giriprakash, Dietmar Cordes, Jessica ZK Caldwell

**Affiliations:** ^1^ Cleveland Clinic, Las Vegas, NV, USA; ^2^ University of Nevada Las Vegas, Las Vegas, NV, USA; ^3^ Cleveland Clinic Lou Ruvo Center for Brain Health, Las Vegas, NV, USA; ^4^ Nevada Exploratory Alzheimer's Disease Research Center, Las Vegas, NV, USA

## Abstract

**Background:**

An estimated 45% of dementia cases are preventable via modifiable risk factors. Our group recently validated the Dynamic Neurocognitive Adaptation (dNA) scale to assess lifetime involvement in Cognitive (COG) Physical (PHY), Creative (CRE) and Social (SOC) activities, divided into 7 time‐windows, from childhood (0‐10 years old) to old age (+65 years old), to identify activities and time points to enhance prevention and intervention efforts.

**Method:**

The study included 67 participants; 37 CN, 19 MCI, 11AD from the Center for Neurodegeneration and Translational Neuroscience who underwent neuropsychological (NP) assessments. Automated measurements of the cortical thickness (CT) of 34 regions within both the left and right hemispheres were obtained using FreeSurfer v 7.3.2. dNA scale scores across the 4 domains and 7 time‐windows were also collected. Hierarchical multiple regression (HMR) analyses were performed to examine the effect of the domains and time‐windows on NP and CT measurements, controlling for age and education. Models used Bonferroni corrections to reduce Type I error. For all significant results, partial correlations were used to estimate the association between dNA and NP and dNA and CT measurements while controlling for age and education.

**Result:**

The HMR model results indicated that the combination of COG‐1, SOC‐4 and PHY‐6 are good predictors of verbal fluency (*p* < .001). Significant partial correlations were found between COG‐1 (*p* = .001) and PHY‐6 (*p* = .012) and verbal fluency. For the CT, different domains and different time‐windows of the dNA, particularly the COG and CRE domains and time‐windows 1 and 6 were associated with thicker left middle temporal cortex and left superior temporal, and right fusiform, right lateral occipital, and right superior temporal cortex. PHY and SOC in time‐windows 1, 4, and 6, were also associated with thicker regions, but with less magnitude.

**Conclusion:**

Cognitive, physical, creative and social activities are protective factors against cognitive impairment and AD. Our results show associations between involvement degree in these activities with NP and CT. Thicker brain (neuro) regions associated with the dNA are consistent with neuropsychological (cognitive) associated with the scale, showing the opportunity of the dNA to capture a neuro‐cognitive adaptation mechanisms through life.

